# Cerebral oxygenation and body mass index association with cognitive function in chronic kidney disease patients without dialysis: a longitudinal study

**DOI:** 10.1038/s41598-022-15129-2

**Published:** 2022-06-25

**Authors:** Susumu Ookawara, Kiyonori Ito, Yusuke Sasabuchi, Mayako Miyahara, Tomoka Miyashita, Nana Takemi, Chieko Nagamine, Shinobu Nakahara, Yuko Horiuchi, Nagisa Inose, Michiko Shiina, Miho Murakoshi, Hidenori Sanayama, Keiji Hirai, Yoshiyuki Morishita

**Affiliations:** 1 Division of Nephrology, First Department of Integrated Medicine, Saitama Medical Center, Jichi Medical University, 1-847 Amanuma-cho, Omiya-ku, Saitama, Saitama 330-8503 Japan; 2 Data Science Center, Jichi Medical University, Tochigi, Japan; 3 Department of Nutrition, Saitama Medical Center, Jichi Medical University, Saitama, Japan; 4 Division of Neurology, First Department of Integrated Medicine, Saitama Medical Center, Jichi Medical University, Saitama, Japan

**Keywords:** Risk factors, Prognostic markers, Chronic kidney disease

## Abstract

In chronic kidney disease (CKD) patients, the prevalence of cognitive impairment increases with CKD progression; however, longitudinal changes in cognitive performance remain controversial. Few reports have examined the association of cerebral oxygenation with cognitive function in longitudinal studies. In this study, 68 CKD patients were included. Cerebral regional oxygen saturation (rSO_2_) was monitored. Cognitive function was evaluated using mini-mental state examination (MMSE) score. Clinical assessments were performed at study initiation and 1 year later. MMSE score was higher at second measurement than at study initiation (p = 0.022). Multivariable linear regression analysis showed that changes in MMSE were independently associated with changes in body mass index (BMI, standardized coefficient: 0.260) and cerebral rSO_2_ (standardized coefficient: 0.345). This was based on clinical factors with p < 0.05 (changes in BMI, cerebral rSO_2_, and serum albumin level) and the following confounding factors: changes in estimated glomerular filtration rate, hemoglobin level, proteinuria, salt and energy intake, age, presence of diabetes mellitus, history of comorbid cerebrovascular disease, and use of renin–angiotensin system blocker. Further studies with a larger sample size and longer observational period are needed to clarify whether maintaining BMI and cerebral oxygenation improve or prevent the deterioration of cognitive function.

## Introduction

In chronic kidney disease (CKD) patients, discussion and decisions about healthcare management, including the choice of renal replacement therapy, is important with patients’ appropriate judgements throughout an accurate comprehension and cognitive function^1^. Furthermore, cognitive impairment was reported to affect the decision-making capacity in patients with advanced CKD^2^; and therefore, efforts to maintain cognitive function are essential in the clinical settings of CKD therapy. In previous studies, cognitive impairment was reportedly associated with various clinical factors, such as ageing, classical vascular factors, nephrogenic factors, and a decrease in the estimated glomerular filtration rate (eGFR)^3–7^. In particular, the prevalence of cognitive impairment reportedly increased with a reduction in eGFR^7^. However, it was recently reported that CKD progression and the degree of eGFR reduction are not associated with an increased risk of cognitive impairment^8^. Further, longitudinal studies have shown that cognitive performance was well-maintained in patients with CKD, which could potentially be attributed to specific CKD therapies^2,9,10^. Therefore, the association between changes in cognitive function and clinical factors such as eGFR, and longitudinal changes in cognitive impairment in patients with CKD, remain controversial.

Cerebral regional oxygen saturation (rSO_2_), a real-time marker of cerebral oxygenation, has been evaluated using near-infrared spectroscopy (NIRS) in the clinical setting^11–14^. In particular, CKD patients without dialysis therapy presented with relatively low cerebral rSO_2_ values when compared with normal controls^15–17^, and were reportedly affected by dietary intake and nutritional status, including energy and salt intake^18^. Furthermore, a cross-sectional study demonstrated that there were significant associations between cerebral rSO_2_ and cognitive function^17^, in addition to the association between nutritional status and cognitive function^19^. However, to date, few reports have examined the association of clinical factors, including cerebral oxygenation and nutritional status, with cognitive function in longitudinal studies of CKD patients. Therefore, in this study, we aimed to longitudinally investigate the association between cognitive assessment scores and clinical factors, including cerebral rSO_2_ and nutritional status, in CKD patients without dialysis therapy.

## Results

**Figure 1:**
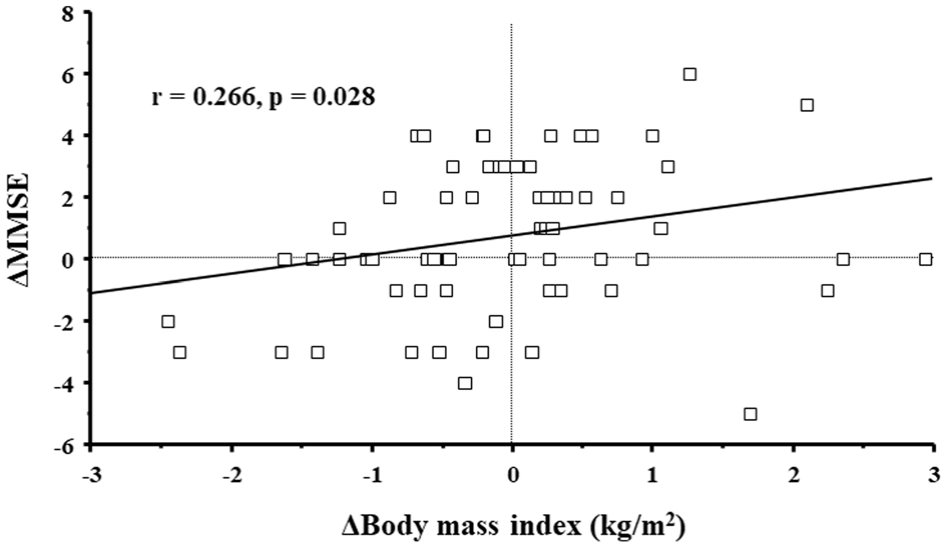
Correlation between changes in MMSE score and changes in body mass index from the study initiation to the second measurement. *ΔMMSE* MMSE score at the second measurement − MMSE score at the study initiation, *ΔBody mass index* body mass index at the second measurement − Body mass index at the study initiation, *MMSE* Mini-Mental State Examination.

**Figure 2:**
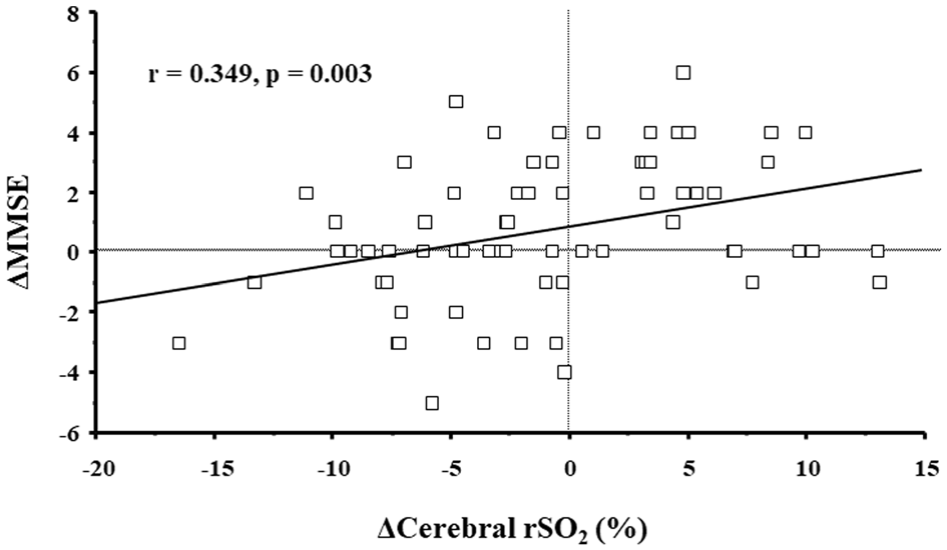
Correlation between changes in MMSE score and changes in cerebral rSO_2_ from the study initiation to the second measurement. *ΔMMSE* MMSE score at the second measurement − MMSE score at the study initiation, *ΔCerebral rSO*_*2*_ cerebral rSO_2_ at the second measurement − cerebral rSO_2_ at the study initiation, *MMSE* Mini-Mental State Examination, *rSO*_*2*_ regional oxygen saturation.

The differences between clinical parameters measured at the study initiation and second measurement are summarized in Table 1. The median follow-up period was 364 days (interquartile range 352–385 days). MMSE score was significantly higher at the time of second measurement than at the study initiation (p = 0.022). The prevalence of cognitive impairment based on the MMSE score decreased at second measurement although the difference was not significant. The scores for the domains of orientation of place and calculation significantly increased at second measurement compared with those at study initiation (p = 0.020 and 0.046, respectively), while slight and significant decrease in orientation of time was observed (p = 0.030). eGFR significantly decreased (p < 0.001) and proteinuria significantly increased (p = 0.006) at second measurement compared with that at the study initiation. However, no other clinical factors evaluated in this study showed significant differences. Furthermore, factors that correlated with the changes in MMSE were investigated using changes in clinical parameters between study initiation and second measurements (Table 2). Changes in body mass index (BMI) (r = 0.266, p = 0.028, Fig. 1), cerebral rSO_2_ (r = 0.349, p = 0.003, Fig. 2), and serum albumin level (r = 0.268, p = 0.027) were significantly correlated with changes in MMSE. Multivariable linear regression analysis was performed for identifying independent factors associated with changes in MMSE using parameters with p < 0.05 (changes in BMI, cerebral rSO_2_, and serum albumin level), and the following confounding factors: changes in eGFR, hemoglobin, proteinuria, salt intake, and energy intake, age, presence of DM, history of comorbid cerebrovascular disease, and use of renin–angiotensin system blocker. As shown in Table 3, changes in MMSE were independently and significantly associated with changes in BMI (standardized coefficient: 0.260) and cerebral rSO_2_ (standardized coefficient: 0.345).

**Table 1:** Comparison of clinical parameters between the study initiation and second measurements in this study.

Variables	Study initiation	Second measurement	p
**MMSE score (30 points)**	27.0 (26.0–29.8)	28.0 (26.0–30.0)	0.022*
Orientation to time (5 points)	5 (5–5)	5 (5–5)	0.030*
Orientation to place (5 points)	5 (5–5)	5 (5–5)	0.020*
Registration (3 points)	3 (3–3)	3 (3–3)	1.000
Calculation (5 points)	3 (1–5)	3 (3–5)	0.046*
Memory recall (3 points)	3 (2–3)	3 (2–3)	0.071
Language (2 points)	2 (2–2)	2 (2–2)	0.317
Repetition (1 point)	1 (1–1)	1 (1–1)	0.257
Complex commands (6 points)	6 (6–6)	6 (6–6)	0.093
MMSE score ≤ 23	5 (7.4)	2 (2.9)	0.438
Body mass index (kg/m^2^)	23.0 (21.3–26.5)	23.0 (21.7–25.7)	0.452
Mean blood pressure (mmHg)	97.2 ± 14.0	95.5 ± 14.0	0.299
Sat O_2_ (%)	98.0 (98.0–98.0)	98.0 (98.0–98.0)	0.438
Cerebral rSO_2_ (%)	58.8 ± 6.4	57.9 ± 6.1	0.300
**Laboratory findings**			
eGFR (mL/min/1.73 m^2^)	27.6 ± 10.7	25.6 ± 11.5	< 0.001*
Hb (g/dL)	12.3 ± 1.6	12.3 ± 1.8	0.926
Serum albumin (g/dL)	4.0 ± 0.3	4.0 ± 0.4	0.132
Serum Ca (mg/dL)	9.1 (8.8–9.4)	9.0 (8.8–9.3)	0.367
Serum P (mg/dL)	3.6 (3.1–4.0)	3.6 (3.1–4.1)	0.165
Proteinuria (mg/day)	355 (112–840)	436 (105–1253)	0.006*
**Nutritional markers**			
Salt intake (g/day)	5.6 (4.5–7.9)	5.9 (4.8–7.6)	0.680
Energy intake (kcal/kg ideal BW/day)	28.7 ± 3.2	28.7 ± 4.2	0.940
Protein intake (g/kg ideal BW/day)	0.8 ± 0.2	0.8 ± 0.2	0.870

Categorical data are presented as number (%), continuous data are presented as mean ± standard deviation or median and interquartile range. *Statistically significant.

*BW* body weight, *eGFR* estimated glomerular filtration rate, *Hb* haemoglobin, *MMSE* Mini-Mental State Examination, *rSO*_*2*_ regional oxygen saturation.

**Table 2:** Correlation between changes in MMSE and those in clinical parameters during this study.

ΔMMSE	r	P
ΔBody mass index	0.266	0.028*
ΔMean blood pressure	0.098	0.428
ΔSat O_2_	− 0.171^#^	0.164
ΔCerebral rSO_2_	0.349	0.003*
ΔeGFR	− 0.047	0.703
ΔHb	0.216	0.077
ΔSerum albumin	0.268	0.027*
ΔSerum Ca	0.007^#^	0.957
ΔSerum P	0.109^#^	0.376
ΔProteinuria	0.173^#^	0.158
ΔSalt intake	− 0.116	0.345
ΔEnergy intake	0.158^#^	0.197
ΔProtein intake	0.233^#^	0.056

*eGFR* estimated glomerular filtration rate, *Hb* haemoglobin, *MMSE* Mini-Mental State Examination, *rSO*_*2*_ regional oxygen saturation.

*Statistically significant.

^#^Indicates spearman’s rank correlation for skewed distribution of data.

**Table 3:** Multivariable linear regression analysis: independently associated factors of changes in MMSE.

Variables	Standardized coefficient	95% CI	P
**ΔMMSE**			
ΔBody mass index	0.260	0.023–0.088	0.023
ΔCerebral rSO_2_	0.345	0.045–0.208	0.003

*CI* confidential interval, *MMSE* Mini-Mental State Examination, *rSO*_*2*_ regional oxygen saturation.

## Discussion

This longitudinal observational study focuses on the association between changes in cognitive function and clinical factors, including cerebral rSO_2_ and nutritional status, in CKD patients without dialysis therapy. MMSE scores showed slight, but significant increase at second measurement compared with those at study initiation, and changes in MMSE score were independently associated with changes in BMI and cerebral rSO_2_.

Among the several types of cognitive screening tests utilized in the clinical setting, MMSE was reportedly most commonly utilized^20^. In patients with CKD and those undergoing hemodialysis, MMSE was found to be less superior to the Montreal Cognitive Assessment (MoCA) to detect the cognitive impairment^21,22^. In contrast, the evaluation of cognitive function using MMSE confirmed decreased eGFR to be associated with an increased risk of cognitive impairment, similar to the results using MoCA^23^. Both MMSE and MoCA were also reported to be moderately sensitive for evaluating cognitive impairment in poststroke patients^24^. In addition, in CKD patients, there were no significant differences in area under the receiver operating curve for detecting cognitive impairment between MMSE and the Mini-Addenbrooke’s Cognitive Examination^25^. Based on these reports^23–25^, MMSE would be effective as a screen for cognitive impairment in CKD patients; therefore, MMSE was used in this study. At study initiation, MMSE score was 27.0 (26.0–29.8) and the prevalence of cognitive impairment (defined as MMSE score ≤ 23) was 7.4% with eGFR of 27.6 ± 10.7 mL/min/1.73 m^2^. Previous study has reported that MMSE, impaired MMSE (defined as MMSE score ≤ 23), and eGFR value were 25.3 ± 3.7, 22%, and 24 ± 10 mL/min/1.73 m^2^ in CKD patients with frailty, and were 27.3 ± 2.4, 6%, and 25 ± 11 mL/min/1.73 m^2^ in those without frailty^26^. Thus, cognitive function in CKD patients included in this study might correspond to that in CKD patients without frailty^26^.

The prevalence of cognitive impairment increases according to the progression of CKD stages. Risk factors for cognitive decline in CKD patients are proposed as follows: demographic factors (race, older age), vascular factors (hypertension, diabetes mellitus, a history of cardiovascular disease), and nephrogenic factors (uremic toxins, oxidative stress, anemia, albuminuria, inflammation)^[3,4.27,28]^. In addition, a meta-analysis based on six longitudinal studies reported 1.4 times higher likelihood of poor cognition in patients with CKD compared to those without CKD^29^. In contrast, recent reports have noted the preservation or improvement of cognitive function during the observational period in CKD patients without dialysis therapy^2,9,10^. Additionally, renin–angiotensin system blocker was reported to be effective for preventing the progression of cognitive impairment in patients with CKD^28^. In the present study, cognitive function evaluated by MMSE showed slight, but significant improvement during the study period, and this result is consistent with previous reports^2,9,10^.

Regarding the association between cognitive function and clinical factors in CKD patients without dialysis therapy, a positive and independent association was observed between changes in BMI and cognitive function. To date, the association between BMI and cognitive function remains controversial. Initially, obesity was reportedly a higher risk for cognitive impairment, which was explained by hyperinsulinemia, adipokines and cytokines, and traditional vascular risk factors such as hypertension and dyslipidemia^30^. However, an inverse association between BMI and the risk of dementia was recently reported^31^ and BMI was positively and independently associated with the MMSE score^32^. Furthermore, BMI > 25 kg/m^2^ was associated with a lower risk of cognitive impairment both in patients with and without CKD^8,33^, and this relationship became evident in the elderly aged 65 years or older^33^. The age of CKD patients included in this study was 68.8 ± 11.6 years, which was considered to be relatively high. Therefore, the positive association between changes in cognitive function and those in BMI was consistent with that in previous reports ^31–33^ and was similar to the relationship of BMI and the decrease in mortality, known as reverse epidemiology, in patients undergoing hemodialysis (HD)^34^.

Cerebral rSO_2_ values in CKD patients without dialysis therapy (approximately 55–60%) and those undergoing HD (approximately 40–50%) were reportedly lower than those in healthy controls (approximately 70%)^15–18,35^. Furthermore, these values were reportedly associated with eGFR, serum albumin concentration, serum sodium concentration, hemoglobin level, energy intake, and energy intake/salt intake in CKD patients without dialysis therapy^17,18^. In the evaluation of cognitive performance using the MMSE score in patients undergoing HD, cerebral rSO_2_ values in patients with cognitive impairment (MMSE score ≤ 23) were reported to be significantly lower than those with normal cognition (MMSE score ≥ 24), and cerebral rSO_2_ represented a positive and independent association with the MMSE score^36^. Regarding the association of changes in the cerebral blood flow with CKD stages, lower eGFR, which indicates the deterioration of renal function, was independently associated with lower cerebral blood flow^37^. This association was explained by the commonality of vascular injuries and endothelial dysfunction in brain and kidney^38,39^, and vasoconstriction of cerebral vessels induced by the accumulation of vasoactive agents in CKD status^40^. Therefore, decreases in cerebral blood flow could impair microcirculation, leading to deterioration of cerebral oxygenation in CKD patients. In addition, cerebral rSO_2_ showed a positive and significant correlation with MMSE score in a simple linear regression analysis^17^. In this study, changes in MMSE were independently and significantly associated with changes in cerebral rSO_2_. Therefore, based on this result, cerebral oxygenation status could play an important role in maintaining cognitive function in CKD patients without dialysis therapy.

During the study period, eGFR significantly decreased from 27.6 ± 10.7 mL/min/1.73 m^2^ to 25.6 ± 11.5 mL/min/1.73 m^2^. However, there was no significant association between changes in MMSE score and those in eGFR. In the clinical setting of CKD therapy, cognitive impairment was reported to appear early in the course of CKD and was more global and severe in advanced disease stages^5–7^. In contrast, no association between cognitive impairment and an increased risk of CKD progression was confirmed after accounting for traditional risk factors. The lack of association between them could be partially attributed to the difference in severity of CKD stages; that is, studies in patients with preserved kidney function would likely find positive correlations between both kidney function and cognitive decline^41^. Furthermore, a rapid decline in eGFR (> 4 mL/min/1.73 m^2^) had an increased relative risk of cognitive decline and dementia^42^. In CKD patients included in this study, the lack of association between changes in cognitive function and those in renal function could be due to the relatively advanced CKD status and mild eGFR decline (2.0 ± 3.9 mL/min/1.73 m^2^) exhibited by these patients. Regarding the association of dietary intake and nutritional status on cognitive function, it was found that subjects with insufficient energy intake and increased salt consumption presented with a significantly impaired cognitive status as compared to other subjects^19,43^. However, in this study, there was no association between changes in the MMSE score and those in dietary intake, including energy, salt, and protein. This may be attributed to the fact that 46 of 68 patients in this study received nutritional education for CKD therapy at study initiation. Therefore, intake of salt (median 5.6 g/day), energy [28.7 ± 3.2 kcal/kg ideal body weight (BW)/day], and protein (0.8 ± 0.2 g/kg ideal BW/day) were within the recommended diet therapy levels for CKD patients, even at study initiation. Consequently, variation in the levels of each dietary component was found to be small and might not have been responsible for the changes in cognitive function seen in this study. However, adequate dietary intake for CKD management is essential to maintain the nutritional status, including BMI, as it was found to be associated with changes in cognitive function. Therefore, daily dietary intake of certain nutritional components could play an important role in preventing the deterioration of cognitive function in CKD patients.

The present study had several limitations that should be noted. First, the sample size was relatively small. Second, cognitive testing was performed during outpatient care in our hospital. The result of this being that there was limited time available for examining each patient, and only the MMSE could be used to assess cognitive function so as to avoid a delay in outpatient care. Third, albuminuria was reportedly associated with the progression of cognitive impairment as one of nephrogenic factors in patients with CKD^3,27,28^. However, in this study, albuminuria was not measured; therefore, changes in proteinuria, instead of albuminuria, were used as a confounding factor with changes in MMSE and no association was found between them. Therefore, further evaluation, including the assessment of albuminuria as a confounding factor, would be required. Fourth, the observational period in which changes in cognitive function were monitored was around 1 year after study initiation. This was considered to be a relatively short timeframe and it would be preferable to observe these changes for a longer period. Finally, plausible confounding factors, such as low socioeconomic status, low education levels, inflammation, oxidative stress, and vascular calcification, were not considered in this study. Therefore, further studies over a longer observational period are required to investigate the association between cognitive ability, including evaluation of specific aspects of cognition, and various clinical factors that were not considered in this study.

In conclusion, changes in MMSE score were positively and independently associated with changes in BMI and cerebral rSO_2_ in this longitudinal study. Further prospective studies, with a larger number of CKD patients and a longer observational period, are needed to clarify whether maintaining BMI and cerebral oxygenation improves or prevents deterioration of cognitive function in CKD patients without dialysis therapy.

## Materials and methods

### Study design

This study was approved by the institutional review board of Saitama Medical Center, Jichi Medical University (Saitama, Japan: approval numbers, RIN 15-104 and RINS19-HEN007), and was conducted in accordance with the Declaration of Helsinki (2004 Tokyo revision). All patients signed informed consent forms before participation. This study was performed during routine visits to the outpatient department of our hospital. The patients enrolled in this prospective cohort study underwent clinical assessments at study initiation and again 1 year later. Patient recruitment was performed from July 5, 2018 to July 16, 2020, and the study was conducted between July 5, 2018 and September 2, 2021 in our medical center.

### Patients

In this single-center longitudinal observational study, CKD patients who met the following criteria were enrolled: (1) all-stage CKD patients not yet requiring dialysis, who were followed up with by our Division of Nephrology; (2) patients who were older than 20 years; (3) patients who received dietary education and nutritional assessment for CKD management; and (4) patients who underwent 24-h urine collection for the evaluation of salt and protein intake. Patients diagnosed with the following comorbidities were excluded: congestive heart failure, chronic obstructive pulmonary disease, and apparent neurological disorder.

Of the 102 patients screened, 88 met the inclusion criteria and were enrolled for the study. Overall, 20 patients were excluded from the analysis due to lack of data. Ultimately, 68 patients (49 men, 19 women; mean age, 68.8 ± 11.6 years) were included and patients’ data were analyzed in the present study. The patients’ general characteristics at study initiation are summarized in Table 4. In CKD patients included in this study, the numbers of patients at each CKD stage were as follows: G3a, 6; G3b, 20; G4, 35; and G5, 7. Causes of CKD included type 2 diabetes mellitus (27 patients), nephrosclerosis (23 patients), chronic glomerulonephritis (7 patients), and other causes (11 patients).

**Table 4:** Patient characteristics.

Characteristics	Total	Male	Female
n	68 (100)	49 (72)	19 (28)
Age (year)	68.8 ± 11.6	70.4 ± 10.1	64.7 ± 14.6
CKD stages G3a/3b/4/5	6(9)/20(29)/35(52)/7(10)	6(12)/13(27)/28(57)/2(4)	0(0)/7(37)/7(37)/5(26)
Systolic BP (mmHg)	136.2 ± 21.1	136.1 ± 22.6	136.6 ± 17.3
Diastolic BP (mmHg)	77.6 ± 13.9	76.8 ± 12.9	79.8 ± 16.5
Mean BP (mmHg)	97.2 ± 14.0	96.5 ± 14.9	98.8 ± 11.7
**Causes of CKD**			
Diabetes mellitus	27 (40)	23 (47)	4 (21)
Nephrosclerosis	23 (34)	18 (37)	5 (26)
Chronic glomerulonephritis	7 (10)	1 (2)	6 (32)
Others	11 (16)	7 (14)	4 (21)
**Comorbidities**			
Cerebrovascular disease	3 (4)	2 (4)	1 (5)
Cardiovascular disease	6 (9)	3 (6)	3 (16)
**CKD-associated laboratory findings**			
BUN (mg/dL)	29.0 (21.0–41.0)	29.0 (20.5–41.0)	32.0 (21.0–41.0)
Serum creatinine (mg/dL)	1.9 (1.4–2.5)	2.1 (1.5–2.5)	1.6 (1.3–2.8)
eGFR (mL/min/1.73m^2^)	27.6 ± 10.7	28.8 ± 10.7	24.6 ± 10.4
Serum Na (mEq/L)	139 (138–141)	140 (138–141)	139 (137–141)
Serum K (mEq/L)	4.7 ± 0.5	4.7 ± 0.5	4.7 ± 0.7
Serum Cl (mEq/L)	106 ± 3	106 ± 3	107 ± 4
Serum Ca (mg/dL)	9.1 (8.8–9.4)	9.0 (8.8–9.3)	9.2 (8.9–9.4)
Serum P (mg/dL)	3.6 (3.1–4.0)	3.4 (3.0–3.7)	4.0 (3.7–4.6)
Hb (g/dL)	12.3 ± 1.6	12.6 ± 1.5	11.6 ± 1.7
**Antihypertensive medication**			
Renin–angiotensin system blocker	37 (54)	26 (53)	11 (58)
Calcium channel blocker	38 (56)	28 (57)	10 (53)
**Others**			
Vitamin D analogue	9 (13)	6 (12)	3 (16)
Phosphate binder	5 (7)	3 (6)	2 (11)
Erythropoiesis-stimulating agent	9 (13)	6 (12)	3 (16)
Sedative agent	2 (3)	1 (2)	1 (5)

Categorical data are presented as number (%), continuous data are presented as mean ± standard deviation or median and interquartile range.

*BP* blood pressure, *BUN* blood urea nitrogen, *CKD* chronic kidney disease, *eGFR* estimated glomerular filtration rate.

### Evaluation of patient’s renal function

For the classification of CKD stages, renal function was evaluated using eGFR based on the serum creatinine concentration (S-Cr), and eGFR was calculated using Eq. (1)^44^:

1 $$ \begin{aligned} {\text{eGFR }}({\text{mL}}/{\text{min}}/{1}.{\text{73m}}^{{2}} ) = & {194 } \times {\text{ S}} - {\text{Cr}} - {1}.0{94 } \times {\text{ age}} - 0.{287}\;\left( {\text{for men}} \right) \\ {\text{eGFR }}({\text{mL}}/{\text{min}}/{1}.{\text{73m}}^{{2}} ) = & {94 } \times {\text{ S}} - {\text{Cr}} - {1}.0{94 } \times {\text{ age}} - 0.{287 } \times \, 0.{793}\;\left( {\text{for women}} \right). \\ \end{aligned} $$

### Method of nutritional assessment

Patients included in this study were instructed to record the total quantity of food and beverages consumed either by weight or in household measures, and the methods of food preparation. Energy intake was evaluated by dietitians based on each patients 3-day meal record using the fifth edition of the Japanese Standard Tables of Food Composition, published by the Science and Technology Agency of Japan^45^. Furthermore, 24-h urine collection was performed to enable evaluation of urinary protein excretion (g/day), urinary urea nitrogen (UUN) excretion, and urinary Na^+^ excretion. The urine collection method was as follows: collection began in the morning after the first voided urine was discarded. The entire volume of urine was collected in a disposable 3 L container. To avoid the possibility of inadequate urine collection, all patients were trained to properly collect their urine samples and it was emphasized that collection must be initiated at a specific time and completed at the same time the next day. Daily protein and salt intakes were calculated based on the UUN and urinary Na^+^ excretion values obtained from the 24-h urine collection.

Protein intake was calculated using Maroni’s equation^46^, as described in Eq. (2):

2 $$ {\text{Protein intake}}\left( {{\text{g}}/{\text{kg ideal BW}}/{\text{day}}} \right) = \left( {{\text{BW }}\left( {{\text{kg}}} \right) \times 0.0{31} + {\text{UUN}}\left( {{\text{g}}/{\text{day}}} \right)} \right) \times {6}.{25} \div {\text{ideal BW}}\left( {{\text{kg}}} \right). $$

Salt intake was calculated using Eq. (3):

3 $$ {\text{Salt intake }}\left( {{\text{g}}/{\text{day}}} \right) = {\text{urinary Na}}^{ + } {\text{excretion }}\left( {{\text{mEq}}/{\text{day}}} \right) \div {17} $$

In addition, ideal BW was calculated using Eq. (4):

4 $$ {\text{ideal BW }}\left( {{\text{kg}}} \right) = {\text{body height }}\left( {\text{m}} \right) \times {\text{body height }}\left( {\text{m}} \right) \times {22 }\left( {{\text{kg}}/{\text{m}}^{{2}} } \right) $$

### Patient baseline characteristics and clinical laboratory measurements

The patients baseline characteristics and clinical data were collected from their medical charts. Data on the primary disease leading to CKD and the coexistence of comorbid cardiovascular or cerebrovascular diseases were extracted from the patients’ medical records.

Blood pressure (BP) was measured in the sitting position. Blood samples were obtained at ambient temperatures from each patient. Peripheral blood counts and biochemical parameters were evaluated.

### Monitoring of cerebral oxygenation

Cerebral rSO_2_, a marker of cerebral oxygenation, was monitored using an INVOS 5100c saturation monitor (Covidien Japan, Tokyo, Japan). The principles behind rSO_2_ measurement using this monitor have been previously reported^16,47,48^. Briefly, this instrument uses a light-emitting diode to transmit near-infrared light at two wavelengths (735 and 810 nm) and two silicon photodiodes that act as light detectors to measure oxygenated hemoglobin (Hb) and deoxygenated Hb. The ratio of oxygenated Hb signal strength to the total Hb (oxygenated Hb + deoxygenated Hb) signal strength was calculated, and the corresponding percentage was recorded as a single numerical value that represented the rSO_2_^49,50^. All data obtained with this instrument were immediately and automatically stored. Furthermore, the light paths leading from the emitter to the different detectors shared a common part: the 30-mm detector assessed superficial tissue, whereas the 40-mm detector assessed deep tissue. By analyzing the differential signals collected by the two detectors, cerebral rSO_2_ values in the deep tissue were obtained from a distance of 20–30 mm from the body surface^51,52^. These measurements were performed at 6-s intervals.

Before measurement, patients were asked to sit in the chair for at least 5 min, and an rSO_2_ measurement sensor was attached to the patient’s forehead. Thereafter, rSO_2_ was measured at 6-s intervals for 5 min, and the mean value was calculated.

### Cognitive assessment method

Cognitive impairment was confirmed using the mini-mental state examination (MMSE)^53^. This test has been widely used to evaluate global cognitive function in the clinical setting, including CKD patients^23,25,26,54^. The maximum score was 30, and cognitive impairment was defined as a score ≤ 23 in keeping with standard practice^26,55^. In this study, MMSE was conducted simultaneously with the evaluation of cerebral rSO_2_.

### Statistical analyses

Data are expressed as mean ± standard deviation, or median and interquartile range. First, it was determined whether the data showed normal distribution or not. Using the Shapiro–Wilk Test to evaluate each variable, normally distributed or non-normally distributed results were obtained. Paired Student’s t test was used for values showing normal distribution, and Wilcoxon signed-rank test was used for values that did not show normal distribution when comparing clinical parameters between the study initiation and second measurements. The chi-square test was used to assess the differences in the prevalence of cognitive impairment at study initiation and second measurement complemented by an adjusted residual analysis. Correlations between changes in MMSE score and clinical parameters in CKD patients were evaluated using Pearson’s correlation or Spearman’s rank correlation for data with normal and skewed distribution, respectively. Multivariate linear regression analysis was performed to extract independent factors of changes in MMSE score. All analyses were performed using IBM SPSS Statistics for Windows, version 26.0 (IBM, Armonk, NY, USA). P values < 0.05 were considered statistically significant.

## Data Availability

All data analyzed during this study are available within the paper.
